# Nonphosphorylatable Src Ser75 Mutation Increases Ethanol Preference and Consumption in Mice

**DOI:** 10.1523/ENEURO.0418-18.2019

**Published:** 2019-04-05

**Authors:** Goro Kato

**Affiliations:** Department of Biochemistry, Faculty of Medicine, Graduate Faculty of Interdisciplinary Research, University of Yamanashi, Yamanashi 409-3898, Japan

**Keywords:** ethanol drinking behavior, knock-in mice, phosphorylation, Src, unique domain

## Abstract

Src is highly expressed in CNS neurons and contributes not only to developmental proliferation and differentiation but also to high-order brain functions, such as those contributing to alcohol consumption. Src knock-out mice exhibit no CNS abnormalities, presumably due to compensation by other Src family kinases (SFKs), but have a shortened lifespan and osteopetrosis-associated defects, impeding investigations of the role of Src on behavior in adult mice. However, the Unique domain of Src differs from those in other SFKs and is phosphorylated by cyclin-dependent kinase 1 (Cdk1) and Cdk5 at Ser75, which influences its postmitotic function in neurons. Therefore, ethanol consumption in mice harboring nonphosphorylatable (Ser75Ala) or phosphomimetic (Ser75Asp) Src mutants was investigated. Mice harboring the Ser75Ala Src mutant, but not the Ser75Asp mutant, had a higher preference for and consumption of solutions containing 5% and 10% ethanol than wild-type mice. However, plasma ethanol concentrations and sensitivities to the sedative effects of ethanol were not different among the groups. In mice harboring the Ser75Ala Src mutant, the activity of Rho-associated kinase (ROCK) in the striatum was significantly lower and Akt Ser473 phosphorylation was significantly higher than in wild-type mice. These results suggest that Src regulates voluntary ethanol drinking in a manner that depends on Ser75 phosphorylation.

## Significance Statement

Although Src family kinases (SFKs) contribute to behavioral responses to alcohol, only the role of Fyn has been investigated to date. As Src knock-out mice do not exhibit CNS abnormalities, presumably because the knock-out is compensated by other SFKs, but have shortened lifespans and osteopetrosis-associated defects, the influence of Src activity on ethanol preference and consumption was investigated in mice harboring nonphosphorylatable (Ser75Ala) or phosphomimetic (Ser75Asp) Src mutants. Mice harboring the Ser75Ala mutant, but not the Ser75Asp mutant, consumed more ethanol than wild-type counterparts and showed lower Rho-associated kinase (ROCK) activity but greater Akt phosphorylation in the striatum. These findings implicate Src activity in the regulation of ethanol consumption.

## Introduction

Src, a membrane-associated 60-kDa protein tyrosine kinase expressed ubiquitously in mammalian tissues, is a molecular hub for signal transduction pathways that modulate a variety of cellular functions (Thomas and Brugge, 1997; Frame et al., 2002). In the CNS, Src not only regulates cell proliferation and differentiation but also contributes to high-order brain functions, such as those involved in learning, memory, and behavior (Salter and Kalia, 2004; Ohnishi et al., 2011; Repetto et al., 2014). The Src family kinase (SFK) member Fyn, which is highly homologous to Src, is implicated in acute sensitivity to ethanol (Trepanier et al., 2012). Fyn knock-out mice are more sensitive to the sedative effects of ethanol but do not display alcohol-induced tyrosine phosphorylation at NR2B subunits of NMDA receptors (Miyakawa et al., 1997).

Whether other SFKs regulate responses to ethanol is unknown. In contrast to the increase in their sensitivity to ethanol, Fyn knock-out mice do not show altered ethanol consumption (Cowen et al., 2003; Yaka et al., 2003b). However, ethanol self-administration is reduced in rats receiving microinjections of the SFK inhibitor PP2 in the dorsal striatum (Wang et al., 2007). Thus, SFKs other than Fyn may influence ethanol drinking behavior (Ohnishi et al., 2011). For example, the SFK Lyn may regulate the rewarding effects of alcohol, measured in a conditioned place preference paradigm, in which the alcohol reward was higher in Lyn-deficient mice but attenuated in mice overexpressing a constitutively active mutant of Lyn (Gibb et al., 2011).

Neural tissues of the brain and retina express a neuronal form of Src, and CNS neurons in general express high levels of Src with greater activity than in non-neuronal cells (Sorge et al., 1984; Brugge et al., 1985; Cartwright et al., 1987). Nevertheless, Src knock-out mice do not exhibit detectable abnormalities in neural tissues. However, these mice have shorter lifespans and develop osteopetrosis, impeding investigations of Src regulation of behaviors, such as ethanol drinking, in adult mice. Thus, studies of Src knock-out mice have not yielded a full understanding of the physiologic role of Src, and the effects observed may reflect functional compensation by similar tyrosine kinases (Soriano et al., 1991; Lowell and Soriano, 1996).

Src function is regulated by associations with other molecules or by phosphorylation (Shalloway and Shenoy, 1991), and the regulation of tyrosine kinase activity is similar among SFKs (Brown and Cooper, 1996). They share conserved catalytic and regulatory domains, which are repressed and activated by C-terminal phosphorylation and dephosphorylation, respectively (Sicheri and Kuriyan, 1997; Schwartzberg, 1998), and autophosphorylation within the activation loop stabilizes kinase activation (Xu et al., 1999). However, SFKs differ with regard to their N-terminal Unique domains (Amata et al., 2014). Phosphorylation of Ser75 in the Unique domain of Src is performed by cyclin-dependent kinase 1 (Cdk1) in fibroblasts and is associated with Src activation during mitosis (Chackalaparampil and Shalloway, 1988; Morgan et al., 1989; Shenoy et al., 1989). In differentiated striatal neurons (Cartwright et al., 1987) and some tumor cell lines of neuronal origin (Kato and Maeda, 1995), Ser75 is phosphorylated in a mitosis-independent manner, as shown by analyses of tryptic phosphopeptide maps. Cdk5/p35, which has the same consensus sequence as Cdk1, phosphorylates Ser75 in the Unique domain in human Y79 retinoblastoma cells (Kato and Maeda, 1999) and in *in vitro* phosphorylation assays (Pérez et al., 2009). Moreover, Ser75 phosphorylation promotes ubiquitin-mediated degradation of activated Src (Pan et al., 2011). Src phosphorylated at Ser75 by Cdk5 has been shown to increase Rho-associated kinase (ROCK) activity *in vitro* (Tripathi and Zelenka, 2009). Moreover, ROCK activity is higher in the retinas of mice harboring a mutant Src in which Ser75 was changed to Asp (SD) but not when changed to Ala (SA) (Kashiwagi et al., 2017). Thus, Ser75 phosphorylation may play important roles in CNS neurons.

To elucidate the role of Src activity in the CNS, mutant mouse lines were generated that harbor Src with either the SD mutation, mimicking the phosphorylated form (Kashiwagi et al., 2017), or the SA mutation, which lacks the phosphorylation site. These mice were predicted to exhibit detectable abnormalities, as the mutations are within the Unique domain, which shares no sequence similarity with those of other SFKs. The SD mutant mice exhibit enhanced age-related retinal ganglion cell loss, providing new insight into the role of Src in CNS neurons (Kashiwagi et al., 2017). The present study used SD and SA mutant mice to investigate the role of Src in ethanol consummatory behaviors.

## Materials and Methods

### Animal experiments

Animal experiments were approved by the Animal Care and Use Committee of the University of Yamanashi and conducted in accordance with the Guide for Animal Experimentation from the University of Yamanashi. Mice were housed with autoclave-sterilized paper bedding (Japan SLC Inc.) under a 12/12 h light/dark cycle (lights on at 7 A.M.) in a room maintained at 23 ± 2°C with 50 ± 10% humidity and were provided with food (Oriental Yeast) and water ad libitum.

### Generation of SA and SD mice

The SA and SD mutant mice were generated previously (Kashiwagi et al., 2017) by using a gene-targeting procedure to introduce a point mutation into one allele of the c-*src* gene. For this, the targeting vector comprised 9.0 kb of the c-*src* sequence and a 3.4-kb HSV-tk-neo cassette flanked by 3.2-kb duplications containing part of the c-*src* sequence. In the mutant constructs, exon 2 contained the 2-bp SA (TCC→GCG) or SD (TCC→GAC) mutation. Homologous recombination between the targeting vector and the endogenous gene in transfected CCE embryonic stem cells results in one allele harboring the mutation and the selection cassette, which was verified by Southern blot analysis of genomic DNA from clones resistant to G418 (Kato and Maeda, 2003). Heterozygosity for the mutation was confirmed with dot-blot hybridization of allele-specific oligonucleotide probes targeting the wild-type and SA or SD mutant alleles from PCR-amplified DNA, as described previously (Kato and Maeda, 2003). The selection cassette was then excised by homologous recombination within the duplication at one allele. Heterozygous revertants with successful excision of the selection cassette exhibiting only the point mutation were verified by Southern blot analysis (Kato and Maeda, 2003). Allele-specific oligonucleotide probe dot-blot hybridization analyses were performed to confirm the presence of WT and mutant alleles and the absence of the selection marker, as described above. Chimeras, generated by injecting correctly reverted clones into blastocysts, were mated with C57BL/6 mice, and F1 heterozygotes were crossed to yield F2 offspring. Mice were genotyped via PCR amplification of tail DNA and allele-specific oligonucleotide probe dot-blot hybridization, as described above.

Experiments were performed using SA and SD mutant mice backcrossed six generations to the C57BL/6NCrSlc (Japan SLC, Inc.) background. Mutant mice were maintained by mating heterozygous siblings. Mice heterozygous for SA or SD were intercrossed to generate male SA/SA or SD/SD mice and their wild-type (WT/WT) littermates. Only male mice were used for experimental analyses to avoid the potential influence of the estrous cycle or undetermined physiologic conditions in female mice and because there is evidence for sex differences in ethanol drinking behavior (Crabbe et al., 2006).

### Ethanol consumption and preference

Ethanol consumption and preference were measured by a two-bottle choice test based on a previously described method (Boyce-Rustay and Holmes, 2006). Naive male mice (age, 9–17 weeks) were individually housed for 5 d for habituation and then offered a choice between two bottles for three weeks, one containing 5% or 10% (v/v) ethanol and the other containing tap water. Ethanol and water consumption were measured every 3 or 4 d (six times total), with corrections for evaporation and spillage via measurements from two control bottles (one with tap water and the other with the corresponding ethanol solution) in an empty cage (Camp et al., 2011). At the same time points, mouse body weights were measured and the positions (left/right) of the bottles in each cage were alternated to control for any position bias. Food was available ad libitum during the experiments.

The average amounts of ethanol consumption or total ethanol and water intake per kilogram body weight for each mouse were calculated in two ways. First, the total amounts of ethanol consumption or liquid intake per kilogram body weight per measurement period (3 or 4 d) for the mutant mice were normalized to the average for the WT littermate control, which was defined as a value of 1. Second, a putative daily average amount of ethanol consumption or total ethanol and water intake per kilogram body weight was calculated by dividing the relative total amount by the number of days for each of six total measurements. Ethanol preference was determined by dividing the volume of the ethanol solution consumed by the total volume of ethanol and water consumed.

### Taste preference tests

Taste preferences for sweet (sucrose) and bitter (quinine) tastants were assessed via the above-mentioned two-bottle choice paradigm in accordance with that described previously (Salinas et al., 2014). In this case, the naive male mice (age, 9–17 weeks) were offered a bottle containing water and a bottle containing each of the following tastants for 14 d: first was 0.033% (w/v) sucrose, followed by 0.1% (w/v) sucrose, plain water for 5 d, and then 0.02 mM quinine and finally 0.04 mM quinine. The measurements of body weight and water and tastant consumption, the alternation of the bottle positions, and normalization of total sucrose intake relative to the average for 0.033% (w/v) sucrose for the WT littermates were performed as described above, Ethanol consumption and preference.

### Ethanol metabolism

Blood ethanol concentrations were measured according to a protocol described previously (Harris et al., 1995). Naive male mice (age, 9–17 weeks) were given intraperitoneal injections of 20% ethanol (3 g/kg) in sterile PBS. Blood samples (10 μl) were collected from each mouse 1 and 3 h later and immediately added to 200 μl of ice-cold perchloric acid. The blood samples were centrifuged, and the supernatants were added to 0.6 M potassium hydroxide. The neutralized supernatants (pH 5.5) were used to assay the ethanol concentrations via an alcohol dehydrogenase enzymatic UV test kit (ENZYTEC fluid ethanol, catalog E5340; Thermo Scientific).

### Duration of loss of righting response (LORR)

The duration of the LORR was assessed according to a previously described method (Marley et al., 1986). Naive male mice (age, 9–17 weeks) were administered an intraperitoneal injection of 20% ethanol (3 g/kg) in sterile PBS. Mice that were ataxic and lost the righting response within 5 min after the injection were subjected to the LORR test. The mice were placed on their backs in a V-shaped plastic trough, and the duration of the LORR was recorded for 3 h. Mice were judged to have regained the righting response when they could right themselves five times within 30 s.

### ROCK activity assay

The brains of naive male mice (age, 9–17 weeks) were quickly removed and transferred to a metal plate on ice for the dissection of the striatum, as described previously (Spijker, 2011). The tissue samples were homogenized in 50 µl of ice-cold homogenization buffer containing 50 mM Tris (pH 7.4), 10 mM EDTA, 320 mM sucrose, and 1:50 dilutions of phosphatase inhibitor cocktail 2 (catalog P5726; Sigma-Aldrich) and protease inhibitor cocktail (catalog P8340; Sigma-Aldrich). The homogenates were added to equal volumes of 2× lysis buffer [40 mM Tris (pH 7.4) and 2% Triton X-100] and lysed for 15 min on ice. Insoluble material was removed from the lysate by centrifugation at 20,000 × *g* for 40 min at 4°C.

Bradford protein assays (Bio-Rad Laboratories) were used to determine the protein concentration of the lysates. The immunoassay for ROCK activity (CycLex) was conducted in duplicates of equal amounts of protein (3 μg) according to the manufacturer’s instructions. For visualization, an HRP substrate reagent was added to the wells and incubated for 15 min at 25°C. The absorbance was measured with a spectrophotometric microplate reader (SpectraMAX 340; Molecular Devices) at a wavelength of 450 nm. The activity level (mU/mg of tissue protein) was calculated using recombinant Rho-kinase II (CycLex) as a standard. The activity was corrected by subtracting the value from that in the presence of an inhibitor of ROCKs, 10 μM Y-27632 (catalog 688001; Millipore).

For ROCK immunoblotting, whole tissue proteins (10 μg per sample) were resolved on 4–15% SDS-PAGE gels and transferred to 0.2-μm polyvinylidene difluoride membranes via the Trans-Blot Turbo blotting system (Bio-Rad Laboratories). After blocking with 5% nonfat dry milk in Tris-buffered saline plus 0.05% Tween 20 (TBST), the filter was separated into two pieces, one containing ROCK proteins and the other containing β-tubulin. ROCK filters were incubated with an anti-ROCK-1/2 rabbit polyclonal antibody (1:600 dilution, catalog 07-1458, RRID:AB_10561773; Millipore) for 18 h at 4°C and then incubated with an HRP-conjugated anti-rabbit secondary antibody (1:5000 dilution, catalog NA934; GE Healthcare Life Sciences). The membranes containing β-tubulin were incubated with an HRP-conjugated rabbit monoclonal anti-β-tubulin (9F3) antibody (1:2000 dilution, catalog 5346, RRID:AB_1950376; Cell Signaling Technology) for 18 h at 4°C. The blots were visualized with ECL Prime Western blotting detection reagents (catalog RPN2232; GE Healthcare Life Sciences). Images were acquired and quantitated with a LAS 4000 Image Analyzer (GE Healthcare Life Sciences). Only values falling within the linear range were used for quantitative analysis. ROCK protein levels in each tissue were normalized to those of β-tubulin.

### Determination of phosphorylated Akt levels

Striatal tissue homogenates were lysed with equal volumes of 2× RIPA buffer [20 mM Tris (pH 7.2), 300 mM NaCl, 2% Triton X-100, 2% sodium deoxycholate, and 0.2% SDS], and insoluble material was removed by centrifugation at 20,000 × *g* for 40 min at 4°C. Whole tissue proteins (15 μg per sample) were resolved on 4–15% SDS-PAGE gels and transferred to membranes as described in “ROCK activity assay.” After blocking, the membranes were incubated with an anti-phospho-Akt (Ser473) antibody (1:500 dilution, catalog 4058, RRID:AB_331168; Cell Signaling Technology) for 18 h at 4°C and then with the HRP-conjugated anti-rabbit secondary antibody (1:5000 dilution). The blots were visualized, imaged, and quantitated as in ROCK activity assay. The blots were then washed with TBST and immersed in stripping buffer [62.5 mM Tris (pH 6.8), 100 mM 2-mercaptoethanol, and 2% SDS] at 50°C for 45 min with 1 min shaking every 15 min. The blots were washed, and the removal of antibodies was ensured via the absence of an ECL signal. The blots were blocked and incubated with an anti-Akt antibody (1:1000 dilution, catalog 2920, RRID:AB_1147620; Cell Signaling Technology) for 18 h at 4°C and then incubated with an HRP-conjugated anti-mouse secondary antibody (1:5000 dilution, catalog NA931; GE Healthcare Life Sciences). The blots were visualized with ECL Prime Western blotting detection reagents for image acquisition and analysis. Only values falling within the linear range were used for quantitative analysis. Akt phosphorylation levels are expressed as the ratios of phosphorylated Akt to total Akt levels.

### Statistical analysis

The results are presented as mean ± SD. Normally distributed data were analyzed by unpaired two-tailed Welch’s *t* tests or paired factorial ANOVAs followed by Bonferroni’s or Tukey–Kramer *post hoc* tests, and non-normally distributed data were analyzed by Mann–Whitney *U* tests. All statistical analyses were performed using Ekuseru-Toukei 2012 software (Social Survey Research Information); *p* < 0.05 was considered statistically significant. All statistical analyses are detailed in Table 1.

**Table 1.** Statistical table

Figure/table	Data structure	Type of test	Sample size	Statistical data
Fig. 1*A*				
Ethanol consumption				
WT and SA/SA mice (5%)	Normal distribution	Welch *t* test unpaired	WT: *n =* 6, SA/SA: *n =* 7	CI(d): –2.681 and –0.273, *t* = –2.867, *p* = 0.0226
WT and SA/SA mice (10%)	Normal distribution	Welch *t* test unpaired	WT: *n =* 5, SA/SA: *n =* 5	CI(d): –1.730 and –0.258, *t* = –3.562, *p* = 0.0184
WT and SD/SD mice (5%)	Normal distribution	Welch *t* test unpaired	WT: *n =* 7, SD/SD: *n =* 6	CI(d): –1.705 and 0.765, *t* = –0.906, *p* = 0.3960
WT and SD/SD mice (10%)	Non-normal distribution	Mann–Whitney *U* test	WT: *n =* 8, SD/SD: *n =* 8	*U* = 26, *p* = 0.5286
Fig. 1*B*				
Ethanol preference ratio				
WT and SA/SA mice (5%)	Normal distribution	Welch *t* test unpaired	WT: *n =* 6, SA/SA: *n =* 7	CI(d): –0.444 and –0.056, *t* = –2.987, *p* = 0.0179
WT and SA/SA mice (10%)	Normal distribution	Welch *t* test unpaired	WT: *n =* 5, SA/SA: *n =* 5	CI(d): –0.177 and –0.039, *t* = –3.724, *p* = 0.0080
WT and SD/SD mice (5%)	Normal distribution	Welch *t* test unpaired	WT: *n =* 7, SD/SD: *n =* 6	CI(d): –0.311 and 0.187, *t* = –0.588, *p* = 0.5747
WT and SD/SD mice (10%)	Normal distribution	Welch *t* test unpaired	WT: *n =* 8, SD/SD: *n =* 8	CI(d): –0.109 and 0.094, *t* = –0.155, *p* = 0.8791
Fig. 1*C*				
Total liquid intake				
WT and SA/SA mice (5%)	Normal distribution	Welch *t* test unpaired	WT: *n =* 6, SA/SA: *n =* 7	CI(d): –0.130 and 0.296, *t* = 0.916, *p* = 0.3900
WT and SA/SA mice (10%)	Normal distribution	Welch *t* test unpaired	WT: *n =* 5, SA/SA: *n =* 5	CI(d): –0.156 and 0.133, *t* = –0.190, *p* = 0.8546
WT and SD/SD mice (5%)	Normal distribution	Welch *t* test unpaired	WT: *n =* 7, SD/SD: *n =* 6	CI(d): –0.272 and 0.117, *t* = –0.877, *p* = 0.3999
WT and SD/SD mice (10%)	Normal distribution	Welch *t* test unpaired	WT: *n =* 8, SD/SD: *n =* 8	CI(d): –0.223 and 0.191, *t* = –0.166, *p* = 0.8712
Fig. 2*A*				
Taste preference ratio (WT and SA/SA)				
Sucrose	Normal distribution	Paired factorial ANOVA	WT: *n =* 5, SA/SA: *n =* 5	Genotype: *F* = 0.5107, *p* = 0.4951
0.033% (w/v)		Bonferroni’s *post hoc*		CI(m): WT, 0.477 and 0.569; SA/SA, 0.489 and 0.580, *p* = 0.5901
0.1% (w/v)		Bonferroni’s *post hoc*		CI(m): WT, 0.430 and 0.521; SA/SA, 0.447 and 0.538, *p* = 0.4224
Quinine	Normal distribution	Paired factorial ANOVA	WT: *n =* 5, SA/SA: *n =* 5	Genotype: *F* = 0.0331, *p* = 0.8602
0.02 mM		Bonferroni’s *post hoc*		CI(m): WT, 0.134 and 0.522; SA/SA, 0.139 and 0.527, *p* = 0.9484
0.04 mM		Bonferroni’s *post hoc*		CI(m): WT, 0.033 and 0.421; SA/SA, 0.060 and 0.448, *p* = 0.6783
Fig. 2*B*				
Taste preference ratio (WT and SD/SD)				
Sucrose	Normal distribution	Paired factorial ANOVA	WT: *n =* 5, SD/SD: *n =* 5	Genotype: *F* = 0.8475, *p* = 0.3842
0.033% (w/v)		Bonferroni’s *post hoc*		CI(m): WT, 0.495 and 0.604; SD/SD, 0.447 and 0.557, *p* = 0.0533
0.1% (w/v)		Bonferroni’s *post hoc*		CI(m): WT, 0.518 and 0.627; SD/SD, 0.522 and 0.631, *p* = 0.8722
Quinine	Normal distribution	Paired factorial ANOVA	WT: *n =* 5, SD/SD: *n =* 5	Genotype: *F* = 0.4920, *p* = 0.5029
0.02 mM		Bonferroni’s *post hoc*		CI(m): WT, 0.231 and 0.540; SD/SD, 0.190 and 0.499, *p* = 0.4454
0.04 mM		Bonferroni’s *post hoc*		CI(m): WT, 0.056 and 0.365; SD/SD, 0.003 and 0.312, *p* = 0.3294
Fig. 2*C*				
Total sucrose intake				
WT and SA/SA mice	Normal distribution	Paired factorial ANOVA	WT: *n =* 5, SA/SA: *n =* 5	Genotype: *F* = 0.3578, *p* = 0.5663
0.033% (w/v)		Bonferroni’s *post hoc*		CI(m): WT, 0.442 and 1.558; SA/SA, 0.578 and 1.694, *p* = 0.4868
0.1% (w/v)		Bonferroni’s *post hoc*		CI(m): WT, 2.105 and 3.221; SA/SA, 2.258 and 3.375, *p* = 0.4345
WT and SD/SD mice	Normal distribution	Paired factorial ANOVA	WT: *n =* 5, SD/SD: *n =* 5	Genotype: *F* = 0.2553, *p* = 0.6270
0.033% (w/v)		Bonferroni’s *post hoc*		CI(m): WT, 0.442 and 1.558; SD/SD, 0.214 and 1.329, *p* = 0.2855
0.1% (w/v)		Bonferroni’s *post hoc*		CI(m): WT, 2.359 and 3.475; SD/SD, 2.343 and 3.458, *p* = 0.9385
Fig. 3				
Blood ethanol concentration				
WT and SA/SA mice	Normal distribution	Paired factorial ANOVA	WT: *n =* 6, SA/SA: *n =* 5	Genotype: *F* = 0.7817, *p* = 0.3996
1 h		Tukey–Kramer *post hoc*		CI(m): WT, 2085.307 and 2908.035; SA/SA, 2108.846 and 3010.099, *p* = 0.6879
3 h		Tukey–Kramer *post hoc*		CI(m): WT, 1063.610 and 1886.337; SA/SA, 1298.783 and 2200.036, *p* = 0.0947
WT and SD/SD mice	Normal distribution	Paired factorial ANOVA	WT: *n =* 5, SD/SD: *n =* 4	Genotype: *F* = 0.1381, *p* = 0.7212
1 h		Tukey–Kramer *post hoc*		CI(m): WT, 2115.538 and 3687.719; SD/SD, 2109.947 and 3867.699, *p* = 0.7418
3 h		Tukey–Kramer *post hoc*		CI(m): WT, 1211.065 and 2783.246; SD/SD, 769.016 and 2526.767, *p* = 0.2089
Fig. 4				
LORR				
WT and SA/SA mice	Normal distribution	Welch *t* test unpaired	WT: *n =* 13, SA/SA: *n =* 13	CI(d): –12.835 and 11.450, *t* = –0.118, *p* = 0.9072
WT and SD/SD mice	Normal distribution	Welch *t* test unpaired	WT: *n =* 16, SD/SD: *n =* 13	CI(d): –6.790 and 28.367, *t* = 1.261, *p* = 0.2184
Fig. 5*A*				
ROCK activity				
WT and SA/SA mice	Assuming normality	Welch *t* test unpaired	WT: *n =* 4, SA/SA: *n =* 4	CI(d): 18.691 and 158.488, *t* = 3.305, *p* = 0.0228
WT and SD/SD mice	Assuming normality	Welch *t* test unpaired	WT: *n =* 4, SD/SD: *n =* 4	CI(d): –157.109 and 115.935, *t* = –0.421, *p* = 0.6956
Fig. 5*B*				
ROCK protein level				
WT and SA/SA mice	Assuming normality	Welch *t* test unpaired	WT: *n =* 4, SA/SA: *n =* 4	CI(d): –0.015 and 0.014, *t* = –0.141, *p* = 0.8929
WT and SD/SD mice	Assuming normality	Welch *t* test unpaired	WT: *n =* 4, SD/SD: *n =* 4	CI(d): –0.032 and 0.085, *t* = 1.248, *p* = 0.2782
Fig. 5*C*				
ROCK activity/protein.				
WT and SA/SA mice	Assuming normality	Welch *t* test unpaired	WT: *n =* 4, SA/SA: *n =* 4	CI(d): 194.255 and 925.975, *t* = 3.747, *p* = 0.0096
WT and SD/SD mice	Assuming normality	Welch *t* test unpaired	WT: *n =* 4, SD/SD: *n =* 4	CI(d): –1286.927 and 604.178, *t* = –0.937, *p* = 0.3929

Fig. 5*D*				
Phosphorylated Akt				
WT and SA/SA mice	Normal distribution	Welch *t* test unpaired	WT: *n =* 5, SA/SA: *n =* 5	CI(d): –0.077 and –0.007, *t* = –2.751, *p* = 0.0256
Table 2				
Body weight				
Fig. 1 (ethanol two-bottle choice test)				
WT and SA/SA mice (5%)	Normal distribution	Welch *t* test unpaired	WT: *n =* 6, SA/SA: *n =* 7	CI(d): –0.003 and 0.002, *t* = –0.512, *p* = 0.6229
WT and SA/SA mice (10%)	Normal distribution	Welch *t* test unpaired	WT: *n =* 5, SA/SA: *n =* 5	CI(d): –0.002 and 0.009, *t* = 1.561, *p* = 0.1651
WT and SD/SD mice (5%)	Normal distribution	Welch *t* test unpaired	WT: *n =* 7, SD/SD: *n =* 6	CI(d): –0.001 and 0.007, *t* = 1.907, *p* = 0.0874
WT and SD/SD mice (10%)	Normal distribution	Welch *t* test unpaired	WT: *n =* 8, SD/SD: *n =* 8	CI(d): –0.003 and 0.003, *t* = 0.029, *p* = 0.9770
Fig. 2 (taste preference test)				
WT/WT and SA/SA mice	Normal distribution	Welch *t* test unpaired	WT: *n =* 5, SA/SA: *n =* 5	CI(d): –0.005 and 0.007, *t* = 0.435, *p* = 0.6769
WT/WT and SD/SD mice	Normal distribution	Welch *t* test unpaired	WT: *n =* 5, SA/SA: *n =* 5	CI(d): –0.004 and 0.009, *t* = 0.847, *p* = 0.4226
Table 3				
Putative average ethanol consumption				
WT and SA/SA mice				
5% ethanol	Normal distribution	Welch *t* test unpaired	WT: *n =* 6, SA/SA: *n =* 7	CI(d): –2.223 and –0.209, *t* = –2.809, *p* = 0.0240
10% ethanol	Normal distribution	Welch *t* test unpaired	WT: *n =* 5, SA/SA: *n =* 5	CI(d): –1.707 and –0.305, *t* = –3.309, *p* = 0.0107
WT and SD/SD mice				
5% ethanol	Normal distribution	Welch *t* test unpaired	WT: *n =* 7, SD/SD: *n =* 6	CI(d): –1.692 and 0.833, *t* = –0.820, *p* = 0.4414
10% ethanol	Non-normal distribution	Mann–Whitney *U* test	WT: *n =* 8, SD/SD: *n =* 8	*U* = 26, *p* = 0.5286
Table 4				
Putative average total liquid intake				
WT and SA/SA mice				
5% ethanol	Normal distribution	Welch *t* test unpaired	WT: *n =* 6, SA/SA: *n =* 7	CI(d): –19.2958 and 42.1470, *t* = 0.8822, *p* = 0.4074
10% ethanol	Normal distribution	Welch *t* test unpaired	WT: *n =* 5, SA/SA: *n =* 5	CI(d): –12.7726 and 19.1717, *t* = 0.4800, *p* = 0.6468
WT and SD/SD mice				
5% ethanol	Normal distribution	Welch *t* test unpaired	WT: *n =* 7, SD/SD: *n =* 6	CI(d): –29.0687 and 14.5159, *t* = –0.7377, *p* = 0.4766
10% ethanol	Normal distribution	Welch *t* test unpaired	WT: *n =* 8, SD/SD: *n =* 8	CI(d): –25.3723 and 22.8610, *t* = –0.1142, *p* = 0.9111

CI(d), 95% confidence interval for the difference in population means, lower, and upper limits; CI(m), 95% confidence interval for the population means, lower, and upper limits.

## Results

### Ethanol consumption and preference

**Figure 1.**
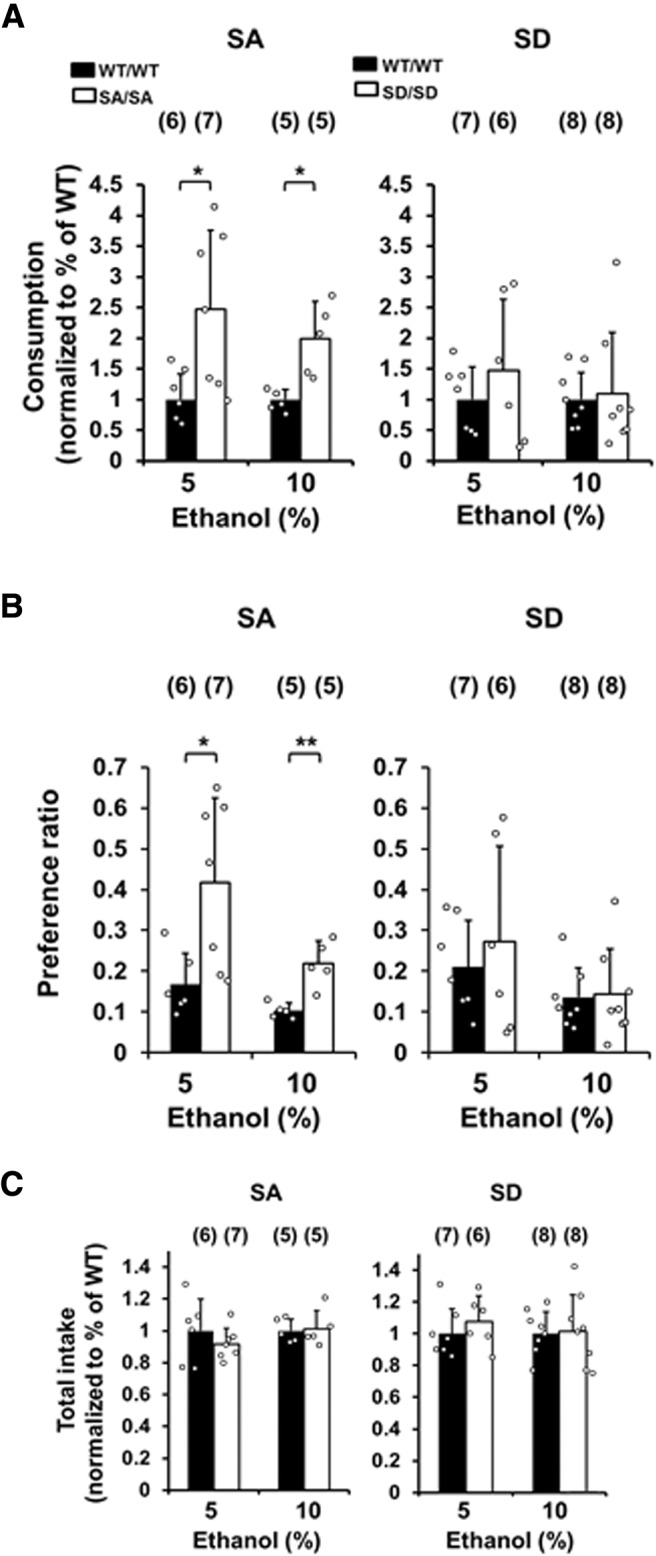
SA/SA mutant mice show increased ethanol consumption. Volitional ethanol intake was measured in a two-bottle choice test between tap water and 5% or 10% ethanol for three weeks in Src Ser75 mutant mice. ***A***, Consumption of a 5% or 10% ethanol solution by SA/SA (left) and SD/SD (right) mice and their WT/WT counterparts. Measurements for mutants were normalized to the average amount per kilogram body weight per measurement period (3 or 4 d) for the WT littermate controls. ***B***, Ethanol preference ratios (volume of ethanol solution consumed per total volume of fluid consumed) of a 5% or 10% ethanol solution in SA/SA (left) and SD/SD (right) mice and their WT/WT counterparts. ***C***, Total fluid intake of a 5% or 10% ethanol solution and water for SA/SA (left) and SD/SD (right) mice and their WT/WT counterparts. Measurements for mutants were normalized to the average amount per kilogram body weight per measurement period for WT littermate controls. Black bars, WT/WT; white bars, SA/SA or SD/SD. Data are mean ± SD. Sample numbers are shown in parentheses; *p < 0.05, **p < 0.01 versus WT/WT by unpaired two-tailed Welch’s *t* tests, except for consumption of 10% ethanol in SD/SD mice, which was analyzed by Mann–Whitney *U* test.

The effect of Src Ser75 phosphorylation on ethanol drinking behaviors was examined in SA and SD mutant mice via a two-bottle choice paradigm. The average ethanol consumption and total ethanol and water intake per measurement period are shown in Figure 1*A*,*C*, respectively. SA/SA mutant mice consumed significantly more ethanol (5% and 10% concentrations) than their WT counterparts (*p* = 0.0226 and *p* = 0.0184, respectively; Fig. 1*A*, left). Consistent with this finding, the preference ratios for 5% and 10% ethanol concentrations were higher for SA/SA mutant mice than for their WT counterparts (*p* = 0.0179 and *p* = 0.0080; Fig. 1*B*, left). By contrast, there were no significant differences between SD/SD mice and their WT counterparts with regard to 5% and 10% ethanol consumption (*p* = 0.3960 and *p* = 0.5286, respectively; Fig. 1*A*, right) and preference (*p* = 0.5747 and *p* = 0.8791, respectively; Fig. 1*B*, right).

The total liquid intake did not differ between SA/SA mice and their WT counterparts (Fig. 1*C*, left) when assessing the 5% ethanol concentration (*p* = 0.3900) or the 10% ethanol concentration (*p* = 0.8546), nor was there a difference between SD/SD mice and their WT counterparts (*p* = 0.3999 and *p* = 0.8712, respectively; Fig. 1*C*, right). There were no significant differences in average body weights between SA or SD mutants and their WT counterparts during the two-bottle choice tests with 5% and 10% ethanol (Table 2).

**Table 2.** Summary of mean body weights during two-bottle choice tests for ethanol and for sucrose or quinine

Experiment	Mean body weight (kg)	*p* value
Fig. 1		
SA		
5% ethanol		
WT/WT	0.028 ± 0.002 (*n =* 6)	
SA/SA	0.028 ± 0.001 (*n =* 7)	0.6229
10% ethanol		
WT/WT	0.031 ± 0.004 (*n =* 5)	
SA/SA	0.028 ± 0.003 (*n =* 5)	0.1651
SD		
5% ethanol		
WT/WT	0.030 ± 0.004 (*n =* 7)	
SD/SD	0.027 ± 0.002 (*n =* 6)	0.0874
10% ethanol		
WT/WT	0.028 ± 0.001 (*n =* 8)	
SD/SD	0.028 ± 0.003 (*n =* 8)	0.977
Fig. 2		
Sucrose/quinine		
SA		
WT/WT	0.036 ± 0.005 (*n =* 5)	
SA/SA	0.035 ± 0.003 (*n =* 5)	0.6769
SD		
WT/WT	0.033 ± 0.004 (*n =* 5)	
SD/SD	0.031 ± 0.005 (*n =* 5)	0.4226

Data are mean ± SD; *p* values are comparisons with WT/WT by unpaired two-tailed Welch’s *t* tests.

### Bitter and sweet taste preferences

**Figure 2.**
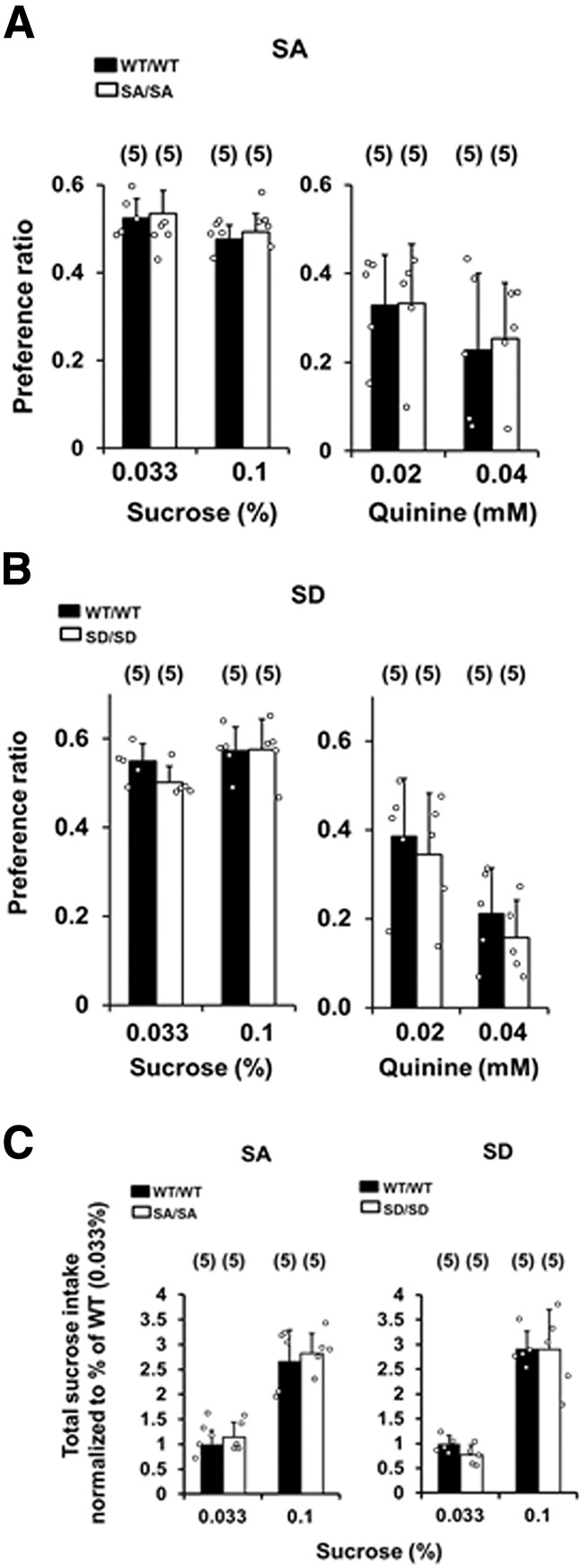
Taste preference ratios for sucrose and quinine in WT and SA/SA (***A***) and SD/SD (***B***) mutant mice. Taste preference was assessed via a two-bottle choice paradigm with tap water and a tastant, each for 14 d, in the following order: 0.033% (w/v) sucrose, 0.1% (w/v) sucrose, 0.02 mM quinine, and 0.04 mM quinine. ***C***, Total intake of a 0.033% (w/v) or 0.1% (w/v) sucrose solution in SA/SA (left) and SD/SD (right) mice and their WT/WT counterparts. Measurements for mutants were normalized to the average amount of 0.033% (w/v) sucrose per kilogram body weight per measurement period for WT littermate controls. Data are mean ± SD analyzed by paired factorial ANOVA and Bonferroni’s *post hoc* tests.

To assess whether the SA or SD mutations altered the preferences for other tastants, the two-bottle choice test was performed with sucrose or quinine solutions. There were no significant differences between SA/SA (Fig. 2*A*) or SD/SD (Fig. 2*B*) mice and their respective WT counterparts in the preference for a sweet or bitter tastant relative to water [SA/SA, *p* = 0.5901 and *p* = 0.4224 for 0.033% (w/v) and 0.1% (w/v) sucrose, respectively, and *p* = 0.9484 and *p* = 0.6783 for 0.02 M and 0.04 M quinine, respectively; SD/SD, *p* = 0.0533 and *p* = 0.8722 for 0.033% (w/v) and 0.1% (w/v) sucrose, respectively, and *p* = 0.4454 and *p* = 0.3294 for 0.02 M and 0.04 M quinine, respectively]. The total intake of calorific sucrose [0.033% (w/v) and 0.1% (w/v) sucrose solutions] did not differ between SA/SA (Fig. 2*C*, left) or SD/SD (Fig. 2*C*, right) mice and their WT counterparts [SA/SA, *p* = 0.4868 and *p* = 0.4345 for 0.033% (w/v) and 0.1% (w/v) sucrose, respectively; SD/SD, *p* = 0.2855 and *p* = 0.9385 for 0.033% (w/v) and 0.1% (w/v) sucrose, respectively; Fig. 2*C*]. There were no significant differences in average body weight between SA or SD mutants and their WT counterparts during the two-bottle choice tests with sucrose or quinine (Table 2).

### Ethanol metabolism and sedation effects

**Figure 3.**
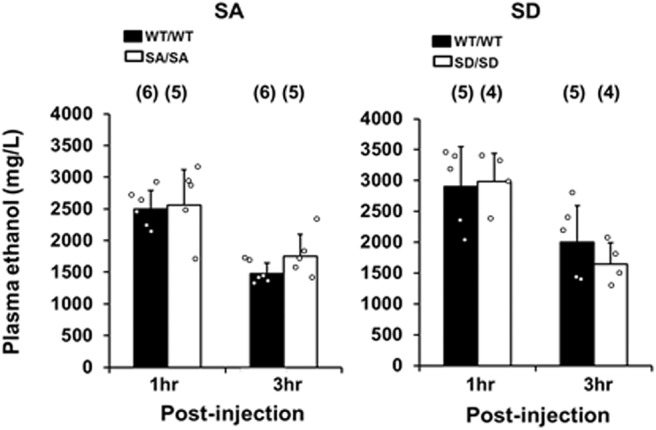
Plasma ethanol levels assayed 1 and 3 h after WT/WT and SA/SA (left) or SD/SD (right) mice were injected with ethanol (3 g/kg). Blood samples (10 μl) were collected from each mouse at the postinjection time points and used for the alcohol dehydrogenase enzymatic spectrophotometric assay. Data are mean ± SD analyzed by paired factorial ANOVA and Tukey–Kramer *post hoc* tests.

The plasma ethanol concentrations at 1 and 3 h after ethanol injections were similar between SA/SA or SD/SD mice and their respective WT counterparts (SA/SA, *p* = 0.6879 and *p* = 0.0949 for 1 and 3 h, respectively; SD/SD, *p* = 0.7418 and *p* = 0.2089 for 1 and 3 h, respectively; Fig. 3). Therefore, SA and SD mutations did not affect ethanol metabolism.

**Figure 4.**
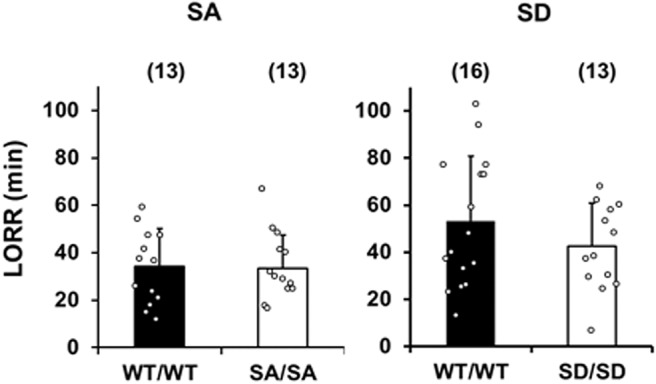
LORR for SA/SA (left) and SD/SD (right) mice and their WT counterparts after they were injected with ethanol (3 g/kg). Data are mean ± SD analyzed by unpaired and two-tailed Welch’s *t* tests.

The sensitivity of SA/SA and SD/SD mice to the sedative effect of ethanol was assessed via the LORR, which has been used to reveal an increased sensitivity to ethanol in Fyn-deficient mice (Trepanier et al., 2012). The responses of mutant mice were similar to those of their WT counterparts (SA/SA, *p* = 0.9072; SD/SD, *p* = 0.1127; Fig. 4).

### Decrease in ROCK activity

**Figure 5.**
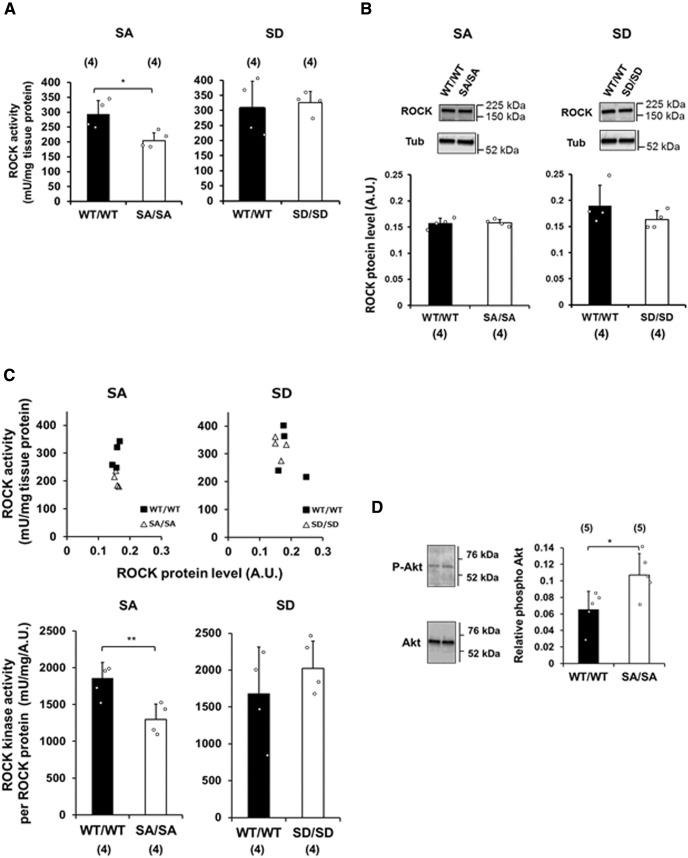
Downregulation of ROCK signaling in striatal tissues from SA/SA mutant mice. ***A***, ROCK activity levels in the striatal tissues of SA/SA (left) and SD/SD (right) mutant mice measured by immunoassays. ***B***, ROCK protein levels in striatal tissues from SA/SA (left) and SD/SD (right) mutant mice measured by Western blotting (upper). ROCK protein level (A.U.) in each tissue was normalized to that of β-tubulin (Tub, lower). ***C***, ROCK kinase activity (mU) per milligram of striatal tissues and normalized to ROCK protein amount (bottom) in SA/SA (left) and SD/SD (right) mutant mice. Scatter diagrams of the two variables are shown in upper panels. ***D***, Phosphorylation of 60-kDa Akt at Ser473 (P-Akt) in the striatal tissues of SA/SA mutant mice as measured by Western blotting. Akt phosphorylation levels were normalized to the level of total Akt. Data are mean ± SD; *p < 0.05, **p < 0.01 by unpaired two-tailed Welch’s *t* tests.

To investigate further why only SA/SA mice but not SD/SD showed higher ethanol preference and consumption despite the shared lack of altered metabolism and sensitivity to ethanol, the phosphorylation of downstream targets was examined. Notably, SD/SD mutant mice exhibit activation of ROCK activity in the retina (Kashiwagi et al., 2017), suggesting that Src Ser75 phosphorylation modulates ROCK as a downstream effector. Thus, ROCK activity was assessed in the striatal tissues of WT and SA/SA mice. ROCK activity was significantly lower (30%) in SA/SA mice than in their WT counterparts (*p* = 0.0228; Fig. 5*A*, left), whereas the activity in SD/SD mice was unaltered (*p* = 0.6956; Fig. 5*A*, right). By contrast, the levels of ROCK protein were similar among the groups (Fig. 5*B*). Accordingly, the level of kinase activity per milligram of protein was significantly lower (30%) in the striatal tissues from SA/SA mice than in their WT counterparts (*p* = 0.0096; Fig. 5*C*, left); ROCK kinase activity per milligram of protein did not differ between SD/SD mice and their WT counterparts (*p* = 0.3929; Fig. 5*C*, right).

To validate these findings, the activation of Akt by ROCK phosphorylation at Ser473 in the activation loop was investigated. In accordance with the observed decrease in ROCK activity, a higher proportion (64%) of Akt was phosphorylated in striatal tissues from SA/SA mutant mice than in those from their WT counterparts (*p* = 0.025; Fig. 5*D*). These results suggest that Akt is activated in the striata of SA/SA mice.

## Discussion

This *in vivo* study demonstrates that a mutation abolishing Src Ser75 phosphorylation increases ethanol preference and consumption in mice. This effect was independent of an alteration of taste perception, as the preference for and consumption of other tastants were not altered. Moreover, the effect of the SA mutation on ethanol consumption cannot be attributed to a difference in ethanol metabolism, because the plasma concentrations in the SA mutants were not different from those in the WT controls. There was also no change in sensitivity to the sedative effects of ethanol as assessed by righting responses.

The average consumption/body weight/d for WT controls (Table 3) was approximately one-quarter to one-fifth of the reported amount for 10% ethanol (Yoshimoto and Komura, 1987), corresponding to an approximately one-third lower preference. This may reflect the differences in the genetic backgrounds or physiologic and environmental factors. Although the various mouse lines were of a C57BL/6N background, genetic polymorphisms can emerge that influence the observed phenotypes. Among 11 C57BL/6N-derived substrains, single nucleotide polymorphisms have been identified at 70% of selected loci that may have interacted with targeted genes and affected the phenotypes in various cases (Mekada et al., 2015). For this reason, SD/SD or SA/SA homozygotes were compared with their WT littermates. Discrepancies among studies may also be attributed to differences in measurement methodologies or environmental conditions, including the types of bedding, diet, cages, and bottles used, which may influence ethanol ingestion behavior or neurotransmitter levels in the brain. Additionally, significant differences in airway responsiveness among some C57BL/6N-derived substrains have been reported, suggesting contributions from both the rearing environment and genetic differences (Chang et al., 2012). Thus, a comparative study of ethanol drinking in different C57BL/6N-derived mouse lines is warranted.

**Table 3.** Daily average ethanol consumption in the two-bottle ethanol choice test

Two-bottle ethanol choice test	Average ethanol consumption (g/kg/d)	*p* value
SA		
5% ethanol		
WT/WT	0.952 ± 0.371 (*n =* 6)	
SA/SA	2.168 ± 1.073 (*n =* 7)	0.0240
10% ethanol		
WT/WT	1.193 ± 0.483 (*n =* 5)	
SA/SA	2.199 ± 0.478 (*n =* 5)	0.0107
SD		
5% ethanol		
WT/WT	0.945 ± 0.487 (*n =* 7)	
SD/SD	1.375 ± 1.201 (*n =* 6)	0.4414
10% ethanol		
WT/WT	1.294 ± 0.565 (*n =* 8)	
SD/SD	1.352 ± 1.197 (*n =* 8)	0.5286

Volitional ethanol intake was measured in a two-bottle choice test between tap water and 5% or 10% ethanol for three weeks in SA and SD mice. The measured values were divided by the number of days for each or the six measurements to obtain putative daily averages. Data are mean ± SD; *p* values are comparisons with WT/WT obtained by unpaired two-tailed Welch’s *t* tests, except for consumption of 10% ethanol in SD/SD mice, which was analyzed by Mann–Whitney *U* test.

In many previous studies, manual 24-h measurements were used to assay ethanol intake in a two-bottle choice test. In this study, manual 3- or 4-d measurements were conducted, although shorter duration measurements could have allowed the detection of more subtle changes; however, manual and shorter duration measurements, which involve changes in bottle position and body weight measurements, are likely to cause stress in mice and affect voluntary drinking. During conventional breeding a mouse consumes 4–6 ml of water per day. The WT controls in this study consumed ∼4 ml. Six male littermates used as WT controls were divided into two groups; one was subject to daily measurements for a total of 7 d, and the other was subject to 3- and 4-d measurements for a total of 7 d to assess voluntary water intake using the same equipment as used in this two-bottle choice study. Water intake per kilogram body weight per 24 h in daily measurements was 133 ± 15 (*n* = 3) and putative daily average intake per kilogram body weight per 24 h in 3- or 4-d measurements was 134 ± 14 (*n* = 3). Moreover, C57BL/6N mice used as WT controls exhibit a 12/12 h light/dark cycle in 12% ethanol intake measured using a fully automated drinkometer system. Clock gene *Period 1* mutant mice lose this circadian rhythmicity and their 24-h intake does not differ from that of WT controls; in addition, intake rhythm and 24-h intakes persist stably across several days (Eisenhardt et al., 2015). Thus, we do not consider that 3- or 4-d measurements are an inaccurate approach. Moreover, the putative daily average amounts of total ethanol and water intake per kilogram body weight (Table 4) were 109–139, while the daily average amounts of total intake per kilogram body weight in daily measurements reported previously were 125–145 in a two-bottle choice paradigm (Salinas et al., 2014) with male C57Bl/6J mice under conditions similar to those described in our study. Taken together, the lower values of ethanol consumption reported in this study do not seem to be due to the longer measurement interval in the two-bottle ethanol choice test.

**Table 4.** Daily average total liquid intake in the two-bottle ethanol choice test

Two-bottle ethanol choice test	Average total intake (g/kg/d)	*p* value
SA		
5% ethanol		
WT/WT	139.3 ± 29.1 (*n =* 6)	
SA/SA	127.9 ± 13.8 (*n =* 7)	0.4074
10% ethanol		
WT/WT	132.1 ± 12.8 (*n =* 5)	
SA/SA	128.9 ± 7.7 (*n =* 5)	0.6468
SD		
5% ethanol		
WT/WT	109.2 ± 17.7 (*n =* 7)	
SD/SD	116.4 ± 17.8 (*n =* 6)	0.4766
10% ethanol		
WT/WT	118.8 ± 15.8 (*n =* 8)	
SD/SD	120.0 ± 26.8 (*n =* 8)	0.9111

Total liquid intake of a 5% or a 10% ethanol solution and water over a period of three weeks by SA and SD mice were measured in the two-bottle choice test. The measured values were divided by the number of days to obtain putative daily averages. Data are mean ± SD; *p* values are comparisons with WT/WT obtained using unpaired two-tailed Welch’s *t* tests.

The lower consumption measured in this study may be subject to a floor effect. However, consumption data from SA/SA mice and their WT controls were normally distributed with a low value for skewness (<0.14), and significant differences were detected for 5% and 10% ethanol. By contrast, data for consumption of 5% ethanol by SD/SD mice were normally distributed but with a higher value for skewness (0.232), which increased to 1.421 for the data for the consumption of 10% ethanol, with a bias toward low values. Ethanol consumption by SD/SD mice did not differ from that by WT controls, but the bias toward lower amounts indicates that lower concentrations (1–3%) should be included in future studies to avoid a floor effect that may mask a detectable difference.

Substrain differences may account for the differences in the LORR duration observed between each mutant strain and the respective WT littermates. The LORR for the WT littermate control of SD mutants was 1.6 times higher than that of WT littermate control of SA mutants. Although both strains were on a C57BL/6NCrSlc (Japan SLC) background, the backcrossing for the SD mutants was performed much earlier than for SA mutants (January 2002 vs May 2005) using the mice obtained from the vendor at those times. Thus, the LORR tests were conducted using SD mice of F16–F17 generations, whereas SA mutants were tested at generations F9–F11. Unknown genetic or environmental factors may have differed for these strains that influenced the observed phenotypes. Nevertheless, it is important to note that the effect of Src gene mutation was isolated by comparing the mutant mice with their respective littermates.

The observed increase in ethanol consumption was not likely motivated by calorie intake, as there was no difference in total sucrose intake or average body weight between SA mutants and their WT counterparts. Pure ethanol has ∼7 kcal/g. Mice fed a diet in which ethanol represented 15% of the total calories exhibited no change in energy intake or expenditure, body weight, or fat content in eight weeks, although they developed hepatic steatosis (Carr et al., 2013). The rodent feed (Oriental Yeast) used in this study contains ∼3.6 kcal/g. WT mice each consumed ∼4 g of feed daily under conventional feeding conditions, representing a daily intake of ∼14 kcal. SA mutant mice each consumed 0.033 g more ethanol daily than their WT counterparts, and the energy intake of the SA mutant mice from ethanol was 0.23 kcal higher than that of their WT counterparts. The increase in energy intake of the SA mutants on an ethanol diet was only ∼2% of the total energy intake of WT mice, implying that the difference in alcohol consumption by the SA mutants was probably not related to calorie intake. Differences in food intake under ad libitum feeding were not evaluated accurately between SA mutants and their WT counterparts, but no major differences were observed. It is unclear whether the SA/SA mutants consumed more ethanol and less food to maintain energy balance. Because SFKs are expressed and function in the arcuate nucleus of the hypothalamus, which is thought to control food intake (Woods et al., 1998), further studies are needed to determine whether SA mutation affects food consumption and energy homeostasis.

Blood ethanol levels following ethanol intake were not assessed in SA mutant mice. However, a previous study found that mice ingesting increased amounts of ethanol did not have higher blood ethanol concentrations in the two-bottle choice test (Olive et al., 2003). Blood concentrations depend on several factors, such as the degree of absorption by digestive organs, whether the ethanol is consumed with or without food, and the diet components. Furthermore, they depend on how the consumption of ethanol is assessed in self-administration tests (Mayfield et al., 2016). Future studies should examine this further, as the relationship between ethanol intake and blood ethanol concentrations is important for investigations of the behavioral effects of alcohol drinking and alcohol abuse disorder in humans (Mayfield et al., 2016).

Of interest is the increase in ethanol consumption in SA mutant mice in the absence of a change in ethanol sensitivity. Previous studies of genetically modified mice have shown a negative relationship between the latency to recover the righting reflex (i.e., sensitivity) and ethanol consumption. For example, PSD-95 knock-out mice have an increased sensitivity to ethanol and decreased ethanol consumption/preference (Camp et al., 2011), whereas neuropeptide Y knock-out mice have a decreased sensitivity and increased consumption/preference (Thiele et al., 1998). Male and female cocaine- and amphetamine-regulated transcript (CART) knock-out mice consume less ethanol than WT counterparts; however, only female CART knock-out mice have an increased sensitivity to ethanol (Salinas et al., 2014). The relationship between ethanol sensitivity and consumption is also less clear for Fyn knock-out mice, which show altered ethanol sensitivity but normal ethanol consumption and reward responses, as measured in the conditioned place preference paradigm (Cowen et al., 2003; Yaka et al., 2003b), but also decreased ethanol preference (Boehm et al., 2003). Thus, a definitive role of Fyn in alcohol drinking behavior remains to be clarified (Ohnishi et al., 2011). Rodent models in some cases have supported this negative relationship between sensitivity and consumption; however, a high sensitivity does not necessarily lead to a low intake of ethanol (Crabbe et al., 2006). Furthermore, long-sleep and short-sleep mice selected according to the duration of alcohol-induced LORR do not differ in alcohol intake and preference (Elmer et al., 1990). Thus, there is no direct correlation between ethanol intake and sensitivity to ethanol (i.e., duration of LORR) in mice. In humans, a low response to modest doses of alcohol is a predictive factor of an increased risk for alcoholism (Schuckit, 1994), and alcoholism has been related to an insensitivity to ataxia and sedation (Krystal et al., 2003). However, individuals with a high or moderate response to consumed alcohol do not always escape alcoholism, because ethanol is addictive. The SA/SA mutant mouse, which exhibits increased preference/consumption despite a moderate sedative response to ethanol, could provide a useful model for studying alcoholism in individuals with a moderate response to modest levels of alcohol drinking. Other measures of alcohol effects, such as motor coordination impairment on tests using rotarods or stationary dowels, can be used to test this hypothesis.

The striatum was chosen for analyses in this study because this brain region is implicated in ethanol drinking and withdrawal in animal models (Chen et al., 2011). Dopamine D2 receptor-mediated signaling in this area correlates with the consumption of ethanol and other addictive substances, such as cocaine and opiates (Kim et al., 2013). D2 receptor stimulation coupled with RhoA/ROCK signaling is also involved in striatal neurodegeneration (Galan-Rodriguez et al., 2017). Notably, ROCK is a downstream effector of Src phosphorylated at Ser 75 (Tripathi and Zelenka, 2009), which can be found in striatal neurons (Cartwright et al., 1987). The SFK inhibitor PP2 microinjected into the striatum decreases ethanol intake in rats (Wang et al., 2007). Thus, the effect of Src phosphorylation on ROCK activity in the striatum was analyzed to identify a molecular mechanism for the increased ethanol intake. The observed increase in alcohol consumption as a result of the SA mutation was accompanied by a decrease in ROCK activation, implicating ROCK as a candidate molecule responsible for the increase in ethanol consumption. Rats given a liquid ethanol diet show reduced ROCK activity in the striatum (Kurt et al., 2015), which suggests that SA mutant mice with constitutively decreased ROCK activity may be predisposed to consume more ethanol. However, genetic gain-of-function studies of ROCK are needed to test this hypothesis. The SA mutation may prolong the half-life of activated Src, as Cdk-dependent phosphorylation promotes its ubiquitin-mediated degradation (Pan et al., 2011). Src kinase activity can suppress the activity of ROCK via direct phosphorylation (Lee et al., 2010) or by the phosphorylation of p190RhoGAP (Brouns et al., 2001). The mice harboring the SD mutation did not consume more ethanol and did not exhibit a decrease in ROCK activity. The present results are in line with previous data showing increased ROCK activity in retinal cells expressing the SD phosphomimetic mutation (Kashiwagi et al., 2017), which likely promotes its degradation and thereby reduces Src activity. Altogether, these data imply that the persistence of active Src affects ethanol consumption/preference.

A decrease in ROCK activity is consistent with the upregulation of Akt phosphorylation in SA mutant mice. ROCK inactivates Akt, a downstream effector in a pathway involving phosphatase and tensin homolog deleted on chromosome 10 (PTEN; Takata et al., 2013). In ROCK/PTEN signaling, RhoA/ROCK phosphorylates PTEN, which enhances its phosphatase activity and stability (Vemula et al., 2010). In turn, PTEN negatively regulates phosphoinositide 3-kinase (PI3K)/Akt signaling by dephosphorylating phosphatidylinositols (PtdIns), reversing the phosphorylation by PI3K (Cantley and Neel, 1999). PI3K also activates Akt; the phosphorylated PtdIns localize Akt to the membrane and open up its catalytic site, resulting in the phosphorylation of Akt at Ser473 and Thr308 in its activation loop by phosphoinositide-dependent kinases. The findings presented here are consistent with those of other studies implicating Akt signaling in alcohol drinking behavior (Neasta et al., 2011; Rao et al., 2015). The results here also suggest that this signaling is regulated by Src, although further studies are needed to verify this as well as to determine how ROCK/Akt signaling in the striatum leads to altered drinking behavior.

Alcoholism is associated with other areas of the mesocorticolimbic dopamine system, such as the intra-ventral tegmental area and nucleus accumbens, as well as the hippocampus, amygdala, and Edinger-Westphal nucleus (Bachtell et al., 2004). These and other brain regions likely cooperate to regulate ethanol drinking behavior. Future studies are needed to determine whether these areas also exhibit Src Ser75 phosphorylation to clarify the molecular mechanism for the Ser75 phosphorylation-dependent increase in ethanol preference. As an intracellular signaling molecule, the effects of Src are pleiotropic, and ROCK is likely not the only key molecule affecting ethanol consumption. Indeed, neurotransmitters, receptors, signaling kinases, and G-proteins are also likely to mediate alcohol drinking behavior. Investigations using Src mutants may help to identify these other contributors.

The results presented here distinguish Src activity from that of the SFK member Fyn, for which there is a large body of evidence demonstrating its influence on acute sensitivity to ethanol. For example, Fyn knock-out mice are more sensitive to the sedative effect of ethanol, with a longer LORR (Miyakawa et al., 1997; Boehm et al., 2003; Yaka et al., 2003b) and impaired motor coordination on a stationary dowel (Boehm et al., 2003). By contrast, mice overexpressing Fyn are less sensitive to the sedative effect of ethanol (Boehm et al., 2004). These effects were attributed to altered tyrosine phosphorylation of the NR2B subunit of NMDA receptors, as knock-out mice did not exhibit the ethanol-induced increase in phosphorylation observed in WT mice (Miyakawa et al., 1997; Yaka et al., 2003a). Although Src and Fyn are the most closely related SFK members and phosphorylate common sites of NMDA receptors, Fyn, but not Src, attenuates the ethanol-induced inhibition of NR1/NR2A-type (but not NR1/NR2B-type) NMDA receptor function in HEK293 cells (Anders et al., 1999a,b). In hippocampal slices exposed to ethanol, the internalization of NR2A-containing (but not NR2B-containing) NMDA receptors was enhanced by the inhibition of Src activity (Suvarna et al., 2005). The ethanol-induced internalization of NR2A-containing receptors may lead to an enrichment of NR2B-containing NMDA receptors at the membrane. The decreased sensitivity and acute tolerance to ethanol could be attributed to NR2B tyrosine phosphorylation by active Fyn; thus, the closely related Fyn and Src kinases differentially regulate NMDA receptor function. Altogether, the evidence suggests that mice with mutations in Src will exhibit different behavioral responses to ethanol. The absence of an effect of the SA or SD mutation on the LORR suggests that these mutations may not directly affect Fyn-mediated NMDA receptor activation. However, to more precisely distinguish Src- and Fyn-mediated functions, it may be necessary to determine whether acute tolerance to ethanol is altered in SA or SD mice via stationary dowel tests of impaired motor coordination, as described previously (Boehm et al., 2003). Furthermore, the SA mutation might affect the ethanol-induced change in the NR2A/NR2B subunit ratio of the membrane NMDA receptor. This should be investigated in a future study. The possibility that Src/ROCK signaling affects NMDA receptor function via actin remodeling cannot be ruled out.

Future analyses will unravel at a molecular level precisely how Src Ser75 phosphorylation regulates volitional ethanol consumption. The involvement of Src Ser75 phosphorylation in addiction, specifically, the reinforcing/rewarding effects of ethanol and abuse/uncontrolled ethanol intake, remains to be clarified. The results presented here suggest that the Src Ser75 mutant mice may be a useful *in vivo* system for behavioral, biochemical, and morphologic studies investigating the regulation of ethanol consumption and for exploring putative targets that may reduce volitional ethanol intake or promote abstinence.

## Synthesis

Reviewing Editor: Karen Szumlinski, University of California at Santa Barbara Decisions are customarily a result of the Reviewing Editor and the peer reviewers coming together and discussing their recommendations until a consensus is reached. When revisions are invited, a fact-based synthesis statement explaining their decision and outlining what is needed to prepare a revision will be listed below.

After consultation on the revised manuscript, we have reached the decision of Revise-Editor Review Only. We agree that the revised manuscript is much improved over the original and that the authors have addressed the large majority of concerns expressed by the two reviewers. However, we agree that the decision to measure alcohol intake every 3-4 days is not an accurate approach to determining alcohol intake. We completely understand that it is difficult for the authors to simply replicate this portion of the study and thus, we ask that they highlight this caveat when they discuss and interpret their findings from the drinking experiment.
